# Tonsilitis*Read before the Brazos Valley Medical Association at Cameron, Texas, May 12 and 13, 1903.

**Published:** 1904-02

**Authors:** W. N. Brooks

**Affiliations:** Franklin, Texas


For Texas Medical Journal.

### Tonsilitis.*

BY W. N. BROOKS, M. D., FRANKLIN, TEXAS.

This paper is gotten up with the view of provoking discussion and as a plea for the conservative treatment of the tonsil rather than destructive.

The tonsil stands at the portal of entrance of the lungs and stomach (at the parting of the ways), and, as is well known, in nature the infinite mind conceives and creates nothing merely as evidence of creative ability, therefore there must be a reason for their creation. In my humble opinion their purpose is to render harmless, as far as possible, the ever present germ outside of contagious diseases, cultures taken from the throat give the streptococcus, staphylococcus, both of which are pathogenic, and there are other less harmful germs present too numerous to count, and, as is well known, only in those who have a lowered resistance from some other cause outside of the throat, usually "cold" or "damp" do we see a follicular attack of tonsilitis. We find tonsilitis in children of weakly constitution either from inheritance or environment. In those with chronic enlarged tonsils, or adenoids, we often have a strumous tubercular or syphilitic parentage. Then, gentlemen, let us, instead of going in and ruthlessly cutting out what was intended to protect the child, treat the tonsil, try to bring it back to a condition of usefulness, bring up the natural resisting power of the body, thereby taking work off the already overworked organ, giving it a chance to get back to a condition where it can fulfill in part or in all the work mapped out for it. Let me make a plea for the doctor to impress on patient and friends the necessity of the doctor seeing a patient after the acute symptoms have passed off until patient's throat and general condition is brought back to a state of health. In the chronic forms local antiseptic astringent with general hygienic and tonic measures will produce a change in the patient's health that will be a surprise to men who think the knife the alpha and omega for these conditions.

As to the relation existing between tonsilitis and rheumatism, might say the cause of rheumatism not being known, it's possible, nay, probable, that the cause is the same, the clinical difference

*Read before the Brazos Valley Medical Association at Cameron, Texas, May 12 and 13, 1903.

caused by difference in absorption or constitutional condition of patient.

To lengthen out this paper and perhaps refresh our memory, we will take the morbid anatomy as given by Osler: "The lacunæ of the tonsils become filled with exudation products, which form cheesy looking masses, projecting from the orifices of the crypts. Not infrequently the exudations from contiguous lacunæ coalesce. The intervening mucosa is usually swollen, deep red in color, and may present herpetic vesicles or, in some instances, even membranous exudations in which case it may be difficult to distinguish the condition from diphtheria. The creamy contents of the crypts are made up of micrococci and epithelial debris."

---

For Texas Medical Journal.

